# EBV VCA IgM and cytomegalovirus IgM dual positivity is a false positive finding related to age and hepatic involvement of primary Epstein–Barr virus infection in children

**DOI:** 10.1097/MD.0000000000012380

**Published:** 2018-09-21

**Authors:** Min Ji Sohn, Jin Min Cho, Jin Soo Moon, Jae Sung Ko, Hye Ran Yang

**Affiliations:** a Department of Pediatrics, Seoul National University Bundang Hospital, Seongnam; b Seoul National University College of Medicine, Seoul, Korea.

**Keywords:** age, child, cytomegalovirus, epstein–barr virus, false positivity, liver

## Abstract

Primary Epstein–Barr virus (EBV) infection is common in childhood, and dual positivity of serum EBV IgM and cytomegalovirus (CMV) IgM antibodies occur in some cases. This study aimed to evaluate the cause of EBV and CMV IgM dual positivity to determine whether it represents a false-positive finding or a true coinfection.

A total of 494 children diagnosed with primary EBV infection, manifesting as infectious mononucleosis, were recruited. The diagnosis was based on positive EBV viral capsid antigen (VCA) IgM antibodies, and serum CMV IgM antibodies and liver enzymes were also evaluated in 149 subjects.

Of 149 children with primary EBV infection, 40 (26.8%) had serum EBV VCA IgM and CMV IgM dual positivity. However, true CMV infection was confirmed only in 1 child of 40 (2.5%) who was positive for both serum CMV Ag and urine CMV polymerase chain reaction (PCR) and negative for serum CMV IgG antibody. Among the children with primary EBV infection, the rate of dual positivity was higher in infants and lower in adolescents (*P* = .013). Liver enzymes were more significantly elevated in children with dual positivity than in those with negative results for CMV IgM antibodies (*P* = .026), which correlated with the serum EBV and CMV IgM titers.

Serum EBV and CMV IgM dual positivity are more prevalent in children with primary EBV infection than what was previously reported. Our results indicate that serum EBV and CMV IgM dual positivity represents a false-positive finding, as opposed to an actual CMV coinfection, possibly due to antigenic cross-reactivity.

## 1 Introduction

Primary Epstein–Barr virus (EBV) infection is a common pediatric infection, and infectious mononucleosis is the most well-known clinical syndrome caused by EBV. It is usually asymptomatic in childhood and manifests in 30%–50% of adolescent cases as the classic triad of fatigue, pharyngitis, and generalized lymphadenopathy.^[1]^

Primary EBV infection is diagnosed by detecting serum IgM antibodies against EBV viral capsid antigen (VCA) in patients suspected of having EBV infection.^[2]^ EBV and cytomegalovirus (CMV) are common opportunistic infections in immunocompromised patients and children.^[3]^ However, dual positivity of serum EBV IgM and CMV IgM antibodies have been reported in some cases of primary EBV infection in children.^[4–8]^ To date, there is a controversy in those cases as to whether dual positivity of EBV IgM and CMV IgM antibodies represents a co-infection of the 2 viruses or a false-positive finding due to the cross-reaction of serum antibodies.^[4–8]^

However, no published reports have investigated the clinical significance of dual positivity of EBV IgM and CMV IgM antibodies in primary EBV infection. In only 1 previous study, EBV and CMV dual positivity was considered indicative of a coinfection or multi-pathogen infection without any evidence based on viral studies.^[3]^ Alternately, other reports have suggested the cross-reaction of serum antibodies as the possible mechanism of dual positive serological reaction in children with primary EBV infection; however, these studies were only case reports or involved adults.^[4–8]^

The present study aimed to demonstrate the clinical features of EBV and CMV IgM dual positivity in children with primary EBV infection and to evaluate the cause of EBV and CMV IgM dual positivity to determine whether it represents a false-positive finding due to cross-reaction or a true coinfection. In addition, based on our findings, we evaluated the association of age and hepatic involvement with positive serum EBV and CMV IgM antibodies in children with primary EBV infection.

## 2 Materials and methods

### 2.1 Patients and data extraction

Among patients presenting to Seoul National University Bundang Hospital from March 2004 through February 2016, 494 pediatric patients aged 18 years or less, who were diagnosed with primary EBV infection manifesting as infectious mononucleosis were initially recruited for the present study. The diagnosis of primary EBV infection was based on positive EBV VCA IgM antibodies and the presence of clinical manifestations compatible with primary EBV infection such as high fever, cervical lymphadenopathy, hepatosplenomegaly, and lymphocytosis with atypical lymphocytes.

We excluded patients who were negative for serum EBV VCA IgM antibodies, those who did not undergo serum CMV IgM antibody tests at the same time or within at least 7 days, those aged < 3 months, and those aged ≥18 years during the clinical course of the primary EBV infection. Patients who were not clinically compatible with primary EBV infection despite positive serum EBV VCA IgM antibodies were also excluded, as well as patients with hepatic dysfunction due to other underlying diseases such as liver diseases, drugs, metabolic disorders, and other systemic infections.

Of the 494 patients, we included only those subjects whose serum CMV IgM antibody levels were measured at the time of blood sampling and evaluated for serum EBV VCA IgM antibodies. A total of 149 children were finally included after a thorough retrospective review of clinical features and laboratory data. The 149 children were classified into 2 groups according to the positivity or negativity of the serum CMV IgM antibodies: the EBV VCA IgM-positive and CMV IgM-negative group (n = 109) and the EBV VCA IgM and CMV IgM dual positive group (n = 40).

The subjects were categorized into 4 age groups according to the Eunice Kennedy Shriver National Institute of Child Health and Human Development guidelines: infant (0–2 years), early childhood (2–5 years), middle childhood (6–11 years), and adolescence (12–18 years).^[9]^

### 2.2 Laboratory tests

From all subjects recruited, routine laboratory tests to determine white blood cell (WBC) count, absolute neutrophil count, lymphocyte count, hemoglobin and hematocrit levels, platelet count, and highly sensitive C-reactive protein levels were performed. Liver function tests for serum aspartate aminotransferase (AST) and alanine aminotransferase (ALT), total and direct bilirubin, and γ-glutamyl transferase were also conducted. Hepatic involvement was defined when serum ALT level was >50 IU/L.

### 2.3 Tests for EBV and CMV infection

Primary EBV infection was defined if there was EBV VCA IgM positivity in the acute phase of the disease. Serum EBV VCA IgM antibody was detected using an enzyme-linked immunosorbent assay (ELISA) in vitro diagnostic kit (Thunderbolt, Gold Standard Diagnostics, CA) for the quantitative measurement of IgM antibodies against the VCA antigens p23 and p18 of EBV. Serum EBV VCA IgM positivity was defined as serum levels above the cutoff value of 0.9 index. Serum EBV VCA IgG antibody was also measured in all subjects, using the same blood samples, and serum EBV early antigen IgM and IgG and EBV Epstein–Barr nuclear antigen IgM and IgG antibodies were checked if available.

Serum CMV IgM antibody was measured using a chemiluminescent microparticle immunoassay (Abbott Corp., IL). The receiver operating characteristic (ROC) curve was developed, and the optimal cutoff for the serum CMV IgM titer was determined using the R program version 3.1.1. The cutoff value of 0.85 index recommended in our tertiary medical center was the optimal cutoff with a sensitivity of 100%, a specificity of 100%, a positive predictive value of 1.0, and a negative predictive value of 1.0, revealing an area under the ROC curve of 1.0. Serum CMV IgM antibody was considered positive when the serum antibody titers were beyond the cutoff value of 0.85 index, and positive CMV IgM antibody result was defined as the dual positivity of serum EBV VCA IgM and CMV IgM antibodies. Serum CMV IgG antibodies were also checked simultaneously.

CMV infection was confirmed when serum CMV antigen and urine CMV polymerase chain reaction (PCR) or culture were found to be positive with simultaneous serum CMV IgM antibody positivity during the acute phase of infectious mononucleosis. Serum CMV antigen assay (Biotest Corp., Dreieich, Germany) was applied using an immunostain with a monoclonal antibody against CMV antigen in blood samples, and the results were expressed as the number of CMV antigen-positive cells per 200,000 leukocytes. The positive result for serum CMV antigen assay was defined as 1 or more CMV antigen positive cell per 200,000 leukocytes. The results of urine CMV PCR or culture were reported as ‘positive’ or ‘negative’ based on the test results. If all tests for CMV were negative, positive CMV IgM antibody result was interpreted as false positivity.

### 2.4 Ethics

This study was conducted with the approval of the Institutional Review Board (IRB) of the Seoul National University Bundang Hospital (IRB No.: B-1701-379-110). Informed consent was formally waived by the IRB.

### 2.5 Statistical analysis

All data are presented as median (range) or as numbers (%). Statistical analysis was performed using SPSS 22.0 statistical software (SPSS Inc., Chicago, IL). Fisher's exact test and χ^2^-square test were used for the statistical analysis of categorical variables to evaluate the differences between the groups. Mann–Whitney *U* test and Kruskal–Wallis test were used for nonparametric statistical analysis of continuous variables between the groups because each group did not show normal distribution. Spearman correlation was used to evaluate the correlation between the 2 continuous variables. Linear regression analysis was also performed to develop a linear model. Scatter plots with a prediction line from a linear model in each group based on the status of CMV IgM positivity were made using the R software program version 3.1.1. The level of statistical significance was set at *P* < .05.

## 3 Results

Clinical features and laboratory findings of the subjects with primary EBV infection and positive EBV VCA IgM antibodies are listed in Table 1. In 149 children with primary EBV infection who underwent tests for CMV IgM antibodies initially, the median age was 7.6 years (ranging from 0.8–17.9 years); 81 patients were boys and 68 were girls. Forty of 149 (26.8%) children showed EBV VCA IgM and CMV IgM dual positivity during the acute phase of the disease, whereas the other 109 children were negative for serum CMV IgM antibody. Only 3 (7.5%) children with serum CMV IgM antibody dual positivity showed urine CMV culture or PCR positivity. Two of those 3 children were also positive for serum CMV IgG antibody and negative for serum CMV antigen while showing low CMV IgM antibody titers. Only 1 patient fulfilled the criteria for a true CMV infection with negative serum CMV IgG antibody and positive serum CMV antigen and urine CMV PCR. The other 37 children with CMV IgM antibody dual positivity had positive serum CMV IgG antibodies and did not show any positive results in the additional testing for CMV infection.

**Table 1:**
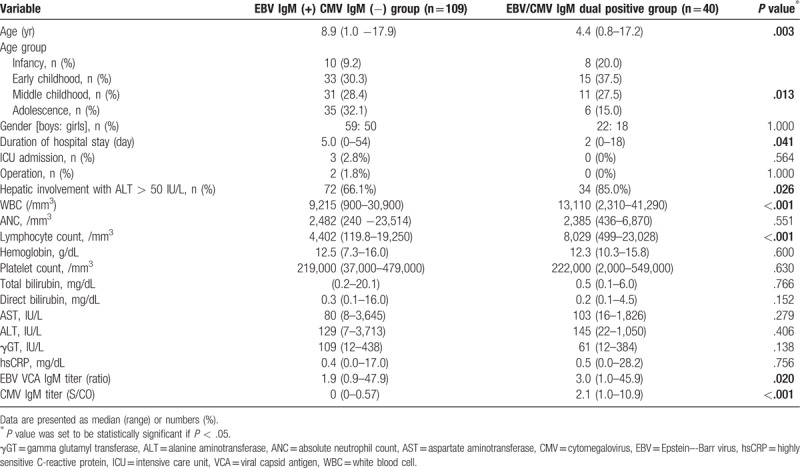
Comparison of clinical features and laboratory parameters between CMV IgM dual positive group and CMV IgM negative group in children with primary EBV infection.

**Figure 1:**
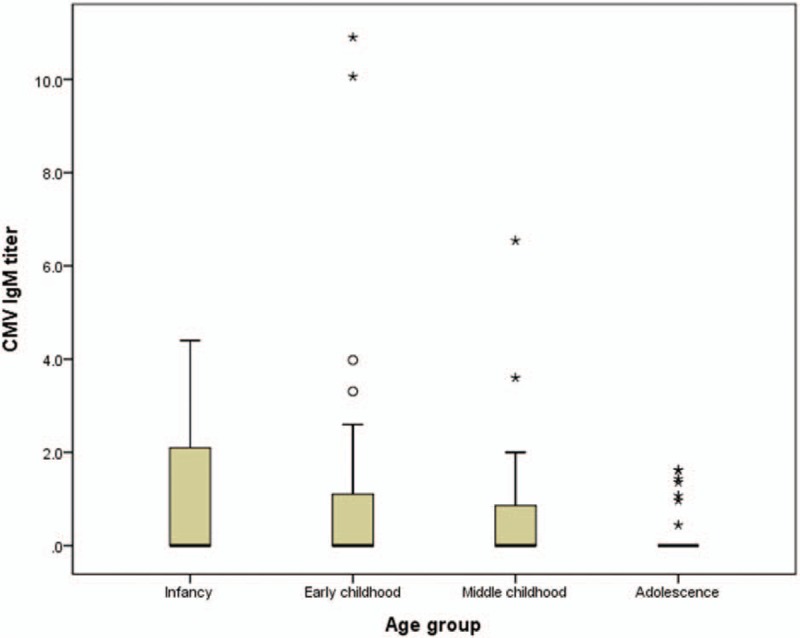
Serum CMV IgM antibody titers were relatively higher in infancy and lower in adolescence in children with primary Epstein-Barr virus infection with serum EBV VCA IgM positivity, but this difference was not statistically significant (*P* = .078). CMV = cytomegalovirus, EBV = Epstein–Barr virus, VCA = viral capsid antigen.

When comparing the clinical features between the 2 groups according to the positivity or negativity of serum CMV IgM antibody in children with primary EBV infection, age was significantly lower in the dual positivity group (*P* = .003) (Table 1). In addition, there was a statistically significant difference between the 4 age groups, in that CMV IgM dual positivity was more prevalent in infancy and less common in adolescence (*P* = .013) (Table 1). Serum CMV IgM antibody titers were relatively higher in infancy and lower in adolescence in children with primary EBV infection with serum EBV VCA IgM positivity, but this difference was not statistically significant (*P* = .078) (Fig. 1).

When laboratory parameters were compared between the EBV VCA IgM-positive and CMV IgM-negative group and the EBV VCA IgM and CMV IgM dual positivity group, the serum EBV IgM titers were significantly higher in the dual positivity group (*P* = .020) (Table 1). WBC and lymphocyte counts were also significantly higher in the dual positivity group (both *P* < .001). In addition, hepatic abnormalities with elevated ALT levels were also more prevalent in the dual positivity group than in the CMV IgM-negative group (*P* = .026) (Table 1).

**Figure 2:**
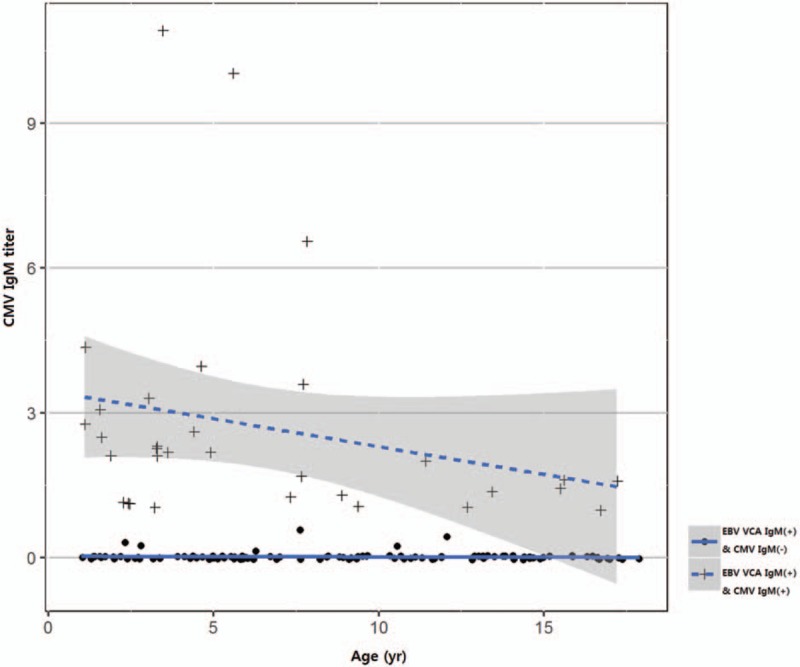
Serum CMV IgM antibody titers negatively correlate with age (*r* = −0.267, *P* = .001) in pediatric patients with primary EBV infection manifested as infectious mononucleosis. CMV = cytomegalovirus, EBV = Epstein–Barr virus.

**Figure 3:**
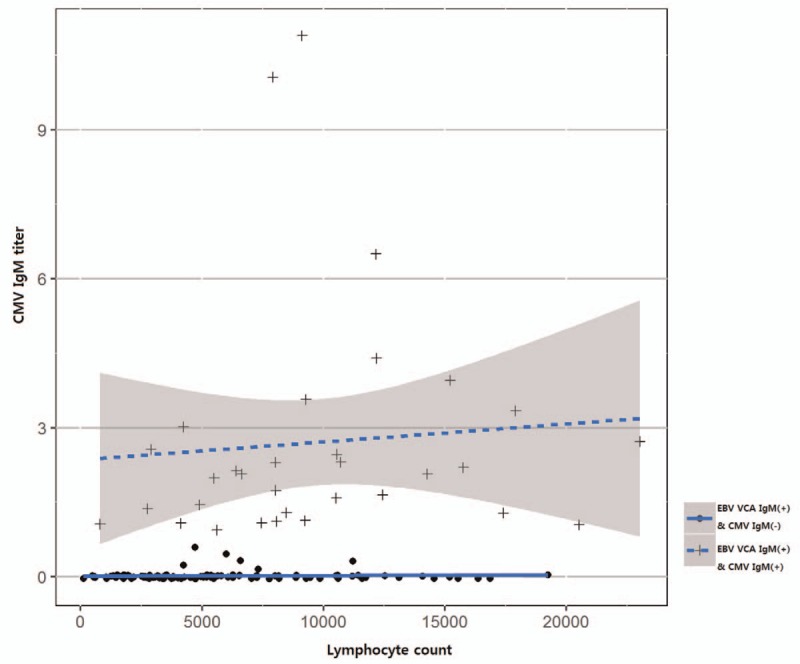
Serum CMV IgM antibody titers positively correlate with lymphocyte counts (*r* = 0.378, *P* < .001) in children with primary EBV infection manifested as infectious mononucleosis. CMV = cytomegalovirus, EBV = Epstein–Barr virus.

As for the correlation between the serum CMV IgM and EBV VCA IgM antibody titers in children with primary EBV infection manifesting as infectious mononucleosis, serum CMV IgM titers showed a significant correlation with serum EBV VCA IgM titers (*r* = 0.245, *P* = .003). Serum CMV IgM antibody titers also showed a significant negative correlation with age (*r* = −0.267, *P* = .001) (Fig. 2), whereas the EBV VCA IgM titers did not (*r* = 0.056, *P* = .496). Among the laboratory parameters, serum CMV IgM antibody titers were significantly correlated with both WBC counts (*r* = 0.326, *P* < .001) and lymphocyte counts (*r* = 0.378, *P* < .001) (Fig. 3). In the EBV VCA IgM-CMV IgM dual positivity group, serum CMV IgM titers were still correlated with lymphocyte counts (*r* = 0.393, *P* = .012) (Fig. 3), but not with WBC counts (*r* = 0.188, *P* = .245).

Regression analysis revealed a significant correlation between serum CMV IgM antibody titers as a dependent factor and age as an independent factor in the process of establishing a linear model (Table 2 and Fig. 2). Regression analysis also showed a significant correlation between the serum CMV IgM titers and lymphocyte counts (Table 2 and Fig. 3).

**Table 2:**
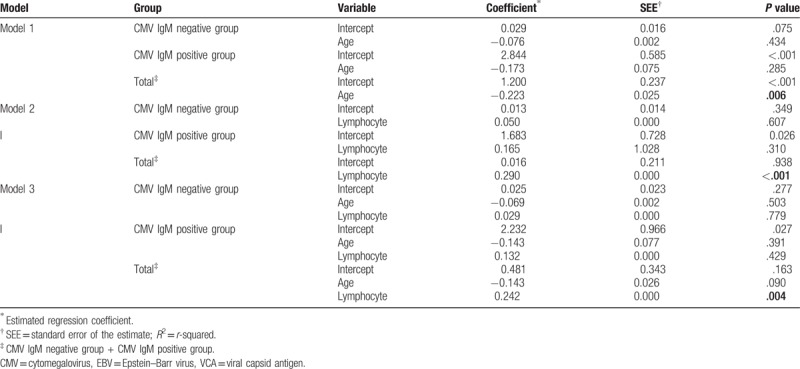
Linear regression modeling for serum CMV IgM antibody titers in children with serum EBV VCA IgM positive primary Epstein-Barr virus infection.

When the subjects were divided into 2 groups according to hepatic involvement, serum EBV VCA IgM and serum CMV IgM titers were both higher in children with hepatic involvement than in those without hepatic involvement (median 2.5, range 0.9–47.9 versus. 1.7, 0.9–7.1; *P* = .006 for EBV VCA IgM; median 0, range 0–10.9 versus 0, 0–3.6; *P* = .006 for CMV IgM) (Fig. 4 A and 4B). Lymphocyte counts were also higher in children with hepatic involvement than in those without hepatic involvement (median 6548.2, range 119.8–23028.0/mm^3^ versus 2833.2, 488.8–17910.0/mm^3^; *P* < .001) (Fig. 4 C).

**Figure 4:**
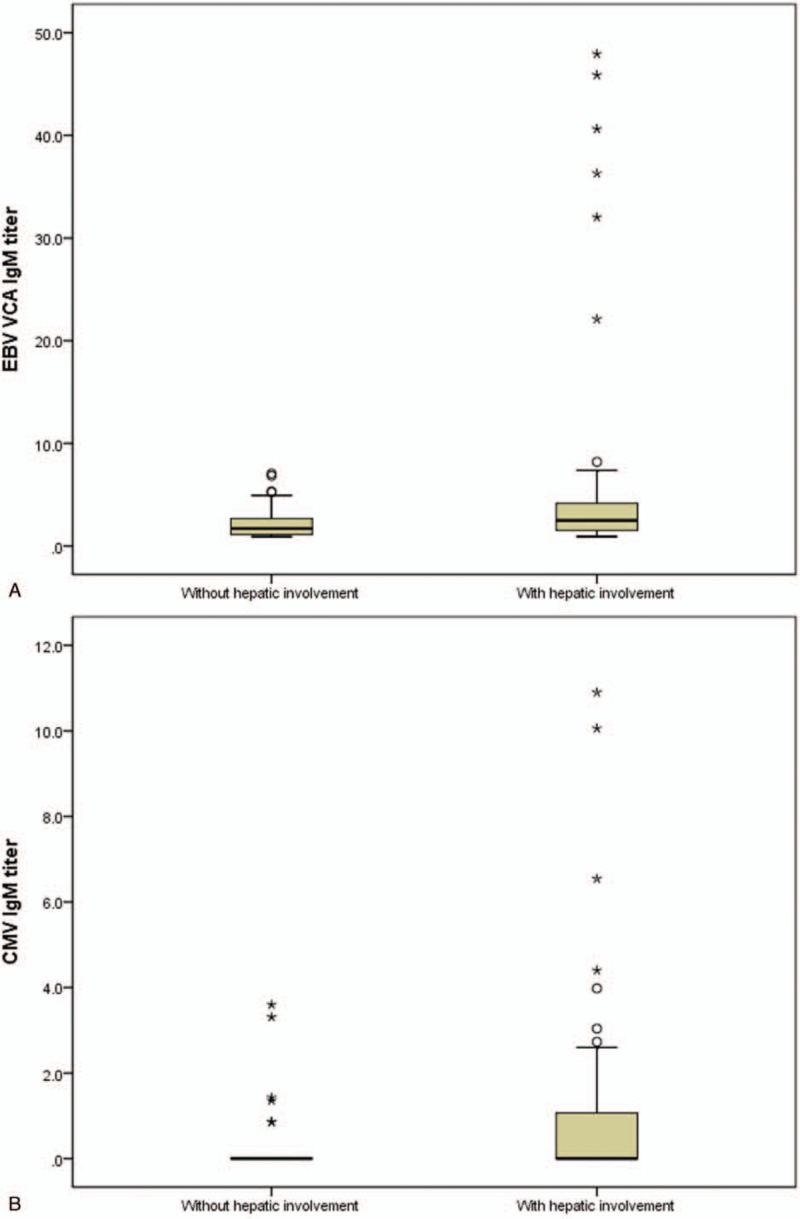
Serum EBV VCA IgM titers (A), CMV IgM titers (B), and lymphocyte counts (C) in children with primary EBV infection were higher in those with hepatic involvement than those without hepatic involvement (*P* = .006 & *P* = .006 & *P* < .001, respectively). CMV = cytomegalovirus, EBV = Epstein–Barr virus, VCA = viral capsid antigen.

**Figure 4 (Continued):**
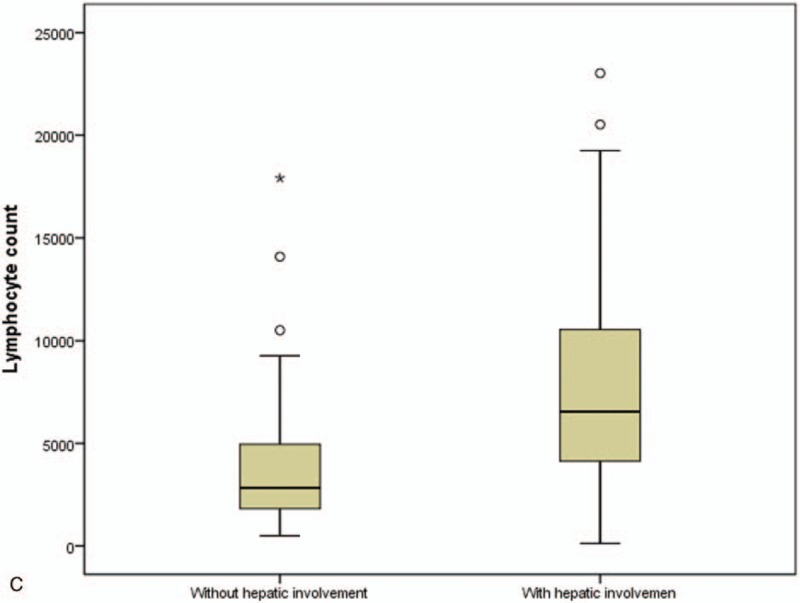
Serum EBV VCA IgM titers (A), CMV IgM titers (B), and lymphocyte counts (C) in children with primary EBV infection were higher in those with hepatic involvement than those without hepatic involvement (*P* = .006 & *P* = .006 & *P* < .001, respectively). CMV = cytomegalovirus, EBV = Epstein–Barr virus, VCA = viral capsid antigen.

## 4 Discussion

This is the first study to evaluate the clinical features and significance of serum EBV and CMV IgM dual positivity in children with primary EBV infection and to investigate its cause to determine whether it is a false-positive finding due to cross-reaction or a representation of actual coinfection with EBV and CMV. Of the 149 children with primary EBV infection, 40 (26.8%) showed serum EBV VCA IgM and CMV IgM dual positivity, but true CMV infection was confirmed only in 1 child (2.5%) with positive serum CMV antigenemia and positive urine CMV PCR in addition to negative serum CMV IgG antibodies.

EBV and CMV are the members of herpes virus family and major cause of common childhood microbial infection.^[3]^ Detection of serum IgM antibodies is suggestive of primary infection with both viruses, but there is wide variability in the sensitivity and the specificity of CMV IgM assays. As CMV IgM antibodies can persist for months after primary infection or reappear during recurrent infection,^[10,11]^ urine CMV culture positivity and positive blood CMV antigen are confirmatory of the diagnosis of true CMV infection in practice.^[12]^ In the present study, among 40 pediatric patients with EBV and CMV IgM dual positivity during the acute phase of infectious mononucleosis, only 3 patients (7.5%) were positive for the additional serum CMV antigenemia assay, urine CMV PCR or urine CMV culture. These 3 patients were all infants aged ≤2.5 years; thus, EBV and CMV coinfection was initially assumed based on the urine CMV PCR or culture positivity accompanied by CMV IgM positivity. However, the CMV IgM titer was very low in 2 of them, along with simultaneous positive serum CMV IgG antibody and negative serum CMV antigen. Consequently, these 2 patients are considered as carriers of CMV infection because CMV IgG positivity is indicative of non-primary infections.^[2]^ True CMV infection was confirmed in only 1 child (2.5%) who was positive for serum CMV antigen urine CMV PCR, and serum CMV IgG antibodies. In the remaining patients, result for CMV IgG was positive, whereas the findings of other CMV tests were negative. Therefore, in light of the false positivity of 39 out of 40 patients revealed in our findings, there is a high probability of serum CMV IgM antibody false positivity caused by a cross-reaction related to the primary EBV infection in cases of EBV and CMV IgM dual positivity.

When we evaluated the clinical factors that could affect the development of EBV and CMV IgM dual positivity in children with primary EBV infection, the age at disease onset was significantly lower in the dual positivity group than in the CMV IgM-negative group. Indeed, the frequency of EBV and CMV IgM dual positivity was significantly higher in infants and lower in adolescents with primary EBV infection in the present study. Moreover, WBC counts and lymphocyte counts were also correlated with serum CMV IgM antibody titers and were significantly higher in the dual positivity group, indicating the significance of the immunologic response in the development of EBV and CMV IgM dual positivity in pediatric patients with EBV-associated infectious mononucleosis.

However, the dual positivity of CMV IgM and EBV VCA IgM during the acute phase of primary EBV infection has been described as a co-infection in previous studies because of the lack of systematized researches.^[13–15]^ Reactivation of EBV and CMV infection due to transient suppression of cellular immunity was found to cause dual positivity in the past,^[16–18]^ even though several pediatric case reports suggested the probability of false positivity in cases of EBV VCA IgM and CMV IgM dual positivity.^[4–8]^ Our study results clearly support the hypothesis that dual positivity might be caused by antigenic cross-reactivity among the herpes viruses, including EBV and CMV, rather than an actual co-infection of these 2 viruses.^[19]^ According to a previous study, EBV virion glycoprotein gp85 was suggested to be immunoprecipitated by anti-sera to CMV and anti-sera to CMV and EBV, neutralizing the infectivity of other herpes viruses at high concentrations.^[19]^ In a previous case report on childhood EBV and CMV IgM dual positivity, this unique finding of dual positivity was explained as selective stimulation of CMV-primed memory B cells by EBV antigen or lymphokines induced by EBV infection or polyclonal B cells stimulated by EBV.^[5]^

In addition, in the present study, serum levels of liver enzymes were significantly elevated in children with EBV and CMV IgM dual positivity compared to those who were negative for CMV IgM, which correlated with the serum EBV and CMV IgM titers. Although hepatic abnormality with elevated serum aminotransferases was more prevalent in children with EBV and CMV IgM dual positivity, serum ALT levels did not directly correlate with serum CMV IgM antibody titers in children with primary EBV. Generally, infectious mononucleosis manifests mostly as the triad of fever, lymphadenopathy, and pharyngitis, and more than 10% of patients present with hepatomegaly and splenomegaly.^[20]^ EBV infection-induced proliferation of lymphocytes and immunoglobulin production may cause lymphadenopathy because of the lymphotropic nature of EBV and its potent B-cell stimulating action.^[5]^ EBV also replicates in the liver similarly to other lymphadenopathy and can cause significant hepatic injuries.^[5]^ Thus, elevated liver enzymes in children with primary EBV infection may not only be a marker for hepatic involvement but also be representative of systemic immunological alterations associated with EBV infection.^[21]^ These changes may enhance the production of immunoglobulin, leading to false-positive serology findings as shown in the present study.

Our study has some limitations. First, only serum EBV VCA IgM-positive patients who underwent simultaneous blood testing for CMV IgM were recruited, leading to the inclusion of only 149 out of 394 patients in the final analysis. Second, a selection bias may have occurred due to the possibility that patients with liver dysfunction were more likely to be selected. Primary EBV infection patients with abnormal liver function test findings or abdominal ultrasonography might have also had their serum CMV IgM antibodies checked. Third, there are the inherent limitations of a retrospective, observational study based on medical records. Further studies are necessary for the future to elucidate the association of age and hepatic involvement with EBV and CMV IgM dual positivity and immunologic responses.

In conclusion, serum EBV VCA IgM and CMV IgM dual positivity in children with primary EBV infection is more prevalent than what was previously reported. Our results indicate that this dual positivity is a false positive finding, possibly due to antigenic cross-reactivity, rather than an indication of coinfection with CMV. Therefore, precautions must be taken in the interpretation of such dual positive results in children with primary EBV infection to prevent unnecessary testing for additional viruses beyond EBV in clinical practice.

## Acknowledgments

The authors are grateful to the Division of Statistics in Medical Research Collaborating Center at Seoul National University Bundang Hospital for statistical analysis. We especially thank Dr Lee JB, a medical statistician, for her help in the statistical analysis and graph designing for this research project.

## Author contributions

**Conceptualization:** Hye Ran Yang.

**Data curation:** Min Ji Sohn, Jin Min Cho.

**Formal analysis:** Jin Soo Moon.

**Investigation:** Min Ji Sohn.

**Supervision:** Jae Sung Ko, Hye Ran Yang.

**Validation:** Hye Ran Yang.

**Visualization:** Hye Ran Yang.

**Writing - original draft:** Min Ji Sohn.

**Writing - review & editing:** Min Ji Sohn.
